# Sexual Dimorphism in the Growth and Morphometric Allometry of the Santandereana Creole Goat Breed in Colombia

**DOI:** 10.3390/vetsci13050501

**Published:** 2026-05-21

**Authors:** Arcesio Salamanca-Carreño, Pere M. Parés-Casanova, Daniel L. Cala Delgado, Jorge L. García Arévalo, Anthony Valverde, Raúl Jáuregui, Mauricio Vélez-Terranova

**Affiliations:** 1 Facultad de Medicina Veterinaria y Zootecnia, Universidad Cooperativa de Colombia, Villavicencio 500001, Colombia; 2 Departamento de Bromatología, Universitat Oberta de Catalunya, 08018 Barcelona, Spain; 3 Grupo de Investigación en Ciencia Animal (GRICA), Facultad de Medicina Veterinaria y Zootecnia, Universidad Cooperativa de Colombia, Bucaramanga 680003, Colombia; 4 Escuela de Ciencias Agrícolas, Pecuarias y del Medio Ambiente, Universidad Nacional Abierta y a Distancia, Bucaramanga 680006, Colombia; 5 Escuela de Agronomía, Laboratorio de Reproducción Animal, Instituto Tecnológico de Costa Rica, Campus San Carlos, Alajuela 223-21002, Costa Rica; 6 Centro Universitario de Oriente, Universidad San Carlos de Guatemala, Chiquimula 20001, Guatemala; 7 Facultad de Ciencias Agropecuarias, Universidad Nacional de Colombia sede Palmira, Palmira 763531, Colombia

**Keywords:** biometry, goat breed, linear characters, local breed, sexual development

## Abstract

Animal growth involves changes in size with age and is associated with environmental factors. The aim was to conduct a study of a representative group of the Santandereana Creole goat breed to evaluate morphometric allometry according to sex. A sample of 23 males and 76 females, ranged 8–72 and 8–84 months of age respectively, was studied. Morphometric allometry was evaluated using 30 linear characters. A Mann–Whitney test assessed right- and left-side differences in horns (length and perimeter) and ears (length and width). A one-way PERMANOVA, using Gower distances, assessed sex differences. Principal component analysis (PCA) using a var-covar matrix identified the most discriminating traits. A one-way ANCOVA, using loadings at PC1, was performed to compare allometric trends between sexes. Differences between sexes were observed in linear traits (*p* = 0.0101) and indices (*p* = 0.0364). No differences were observed between the right and left sides in horns and ears. Differences were observed in size, with body weight, body length, thoracic perimeter, and horn length (right/left) being the most discriminating values reflecting sexual dimorphism of size. The indexes corroborated the larger size of males, especially in relation to skull width, rump length, chest depth, and body weight. Detected differences may reflect variations in the development of skeletal maturity. The indexes confirm that the Santandereana Creole goat is an animal with a tendency for meat production, with larger males and brachycephalic features due to the shape of the head. Discriminatory measures can be considered as parameters of interest for preserving the breed and establishing genetic improvement programs.

## Simple Summary

The Santandereana Creole goat inhabits the department of Santander, Colombia. It is an animal adapted to a rocky, hilly region, feeds on cacti and native grasses, and is part of the culture and tradition and a productive sector for rural communities. To the authors’ knowledge, there are no studies on sexual differentiation in the breed’s growth. A population of 99 animals was studied to evaluate morphometric allometry using 30 linear traits. It was found that sexual differentiation in growth is related to size, with body weight, body length, thoracic perimeter, and horn length being the most discriminating traits. These values were higher in males. Body indexes confirm that the Santandereana Creole goat is an animal with a tendency towards meat production, with larger males and brachycephalic features due to the shape of their heads. The traits that differentiated growth in males and females can be considered of interest for the preservation of the breed and the establishment of genetic improvement programs.

## 1. Introduction

Animal growth involves increases in muscle, bone, organ, and fat deposits and depends on age, size, sex, and hormonal status, and is associated with environmental factors [1]. Body growth in Creole goats is related to the environment in which they have evolved during the adaptation process [2]. Growth and sexual maturity in goats differ significantly between bucklings and doelings, with males generally reaching puberty earlier and experiencing faster growth rates [3].

Allometry refers to the study of how different body parts, organs, or tissues change in size or shape relative to the overall body size [4]. The effect of size on other body traits depends on the effect of body mass on morphological, productive, ecological, evolutionary, and physiological traits [5]. This field is essential for understanding meat production efficiency, breed-specific growth patterns, and sexual dimorphism in domestic breeds. There are some studies on native goats that have evaluated the allometric growth values of various body parts and fat deposits [6], while other studies have evaluated the ontogenetic allometry of body morphology [7].

Body measurements can be used as indicators of animal growth to develop selection criteria [8,9]. Body measurements have been used to quantify body conformation, establish specific measurements and their variations within a breed or population, and understand productive capacities. They help establish genetic relationships and differences between breeds and the influence of the environment [10,11,12,13], to distinguish native goat breeds [14], and to determine sexual dimorphism in body size [15]. Zoometric studies are important because certain morphometric characteristics, for example, body conformation, head size, horns, color, etc., are little influenced by the environment and can provide evidence of animal diversity [16].

Body indexes (zoometric indexes) are relationships between different quantitative morphological variables [17], which, through a statistical study, determine the variability of each of the relationships. Its use lies in the assessment of an ethnological diagnosis or of somatic states that predispose to certain functions and productivity. The indexes are used as classification patterns of different types in which animals can be classified ethnologically [18,19,20]. They are also used to establish phenotypic comparisons between animals of different breeds or to explain their body development [21].

Goat farming (*Capra hircus*) is important for the economy of rural populations around the world, due to their hardiness and they guarantee livestock sustainability in marginal and hard-to-reach regions, taking advantage of low-quality natural resources that are not suitable for other species [22]. They have a per se value in adaptation variables (extreme temperatures, high altitudes, long periods of drought), resistance to diseases and parasites, cultural and religious value, and product quality, among others [23,24,25].

Creole goats are descended from goats that arrived in the Americas from the 16th century onwards, brought by Spanish conquistadors from southern Spain and the Canary Islands [26,27]. The Santandereana Creole goat lives in Colombia. It is a small breed (withers height: 64.8–72 cm; body length: 69.6–77 cm) [28] which is well adapted to dry conditions of the Chicamocha Canyon, where tropical dry forest is dominant. Although a basic morphometric characterization is reported [28], this is the first allometric study on sexual growth differences in this breed. Taking all of this into account, the growth of the kids was studied using different body measurements to determine growth curves and the effect of some non-genetic factors on the goats’ productive performance. This information will allow for the development of tools that can be applied in goat production systems in similar agroecological areas of Colombia. The hypothesis is based on establishing whether there are differences in allometric growth between sexes of the Santandereana Creole goat.

The aim of this cross-sectional descriptive work was to conduct a study of a representative group of this breed to assess the morphometric allometry according to sex. A clearer understanding of growth curves for the breed can help estimate mature weight at an early age, allowing for earlier breeding decisions and increasing the rate of genetic improvement per year.

## 2. Materials and Methods

### 2.1. Study Area

This research was conducted in the Chicamocha Canyon region, Santander Department (Colombia) (6°48′58″ N, 73°300′36″ W; and Altitude: 1540 m). The region has a rainfall range of 700 to 1100 mm, an ambient temperature of 22 to 34 °C and an average relative humidity of 82%. It has semi-desert vegetation, eroded areas with a predominance of slopes, and shrubs and thickets typical of arid climates [29].

### 2.2. Data Source

In this study, 99 Santandereana goats (23 males and 76 females) were measured, ranging in age from 8 to 72 months for males, and from 8 to 84 months for females. The animals came from five different farms located in the Chicamocha Canyon region, Santander Department (Colombia). The farms where the animals were measured belong to cattle farmers associated with the Colombian Association of Santandereana Goat Breeders (with ASOCRIAS being its acronym in Spanish). The farms were selected for convenience after a meeting with the farmers to ensure the availability of infrastructure (handling of “corrals”) for measuring the animals. On each farm, the animals that were available at the time of the visit were measured. All the farms lack a specific management protocol and technology; grazing is extensive, and the animals roam freely across hills and mountains. Some farms have wooden “corrals” where the animals rest at night. The main activity is milk and meat production.

**Figure 1:**
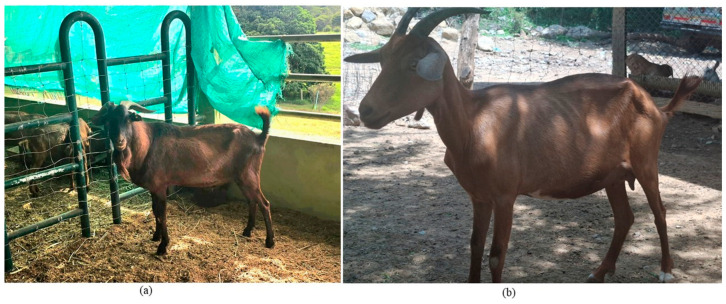
Santandereana Creole goat, Santander department, Colombia. (**a**) Male, (**b**) female. Photograph: Jorge Garcia and Daniel Cala.

The Santanderean Creole goat is adapted to rocky, hillside terrain with high temperatures, constant drought, and low relative humidity. It is small, agile, and resistant to disease (Figure 1). Common coat colors are cherry red and “pejo” (three or more colors). They feed on native grasses of the region (*Melinis minutiflora*, *Aristida adscensionis*, *Panicum maximum*, *Digitaria insularis*, among others), browsing of foliage and fleshy fruits of trees and shrubs such as *Prosopis juliflora* (trupillo tree), *Pithecellobium dulce* (gallinero tree), *Pouteria eugeniifolia* (caimito tree) and Cactaceae such as *Opuntia ficus indica* (“tuna” or nopal), among others. It contributes to the local economy, culture, traditions, and food security of rural communities. The Santandereana Creole goat appears on the DAD-IS website in an “Out of Risk” status, but the population is unknown [30].

### 2.3. Body Measurements

Thirty linear traits were measured in each animal in all specimens (males and females). The methodology used to evaluate the zoometric variables is the commonly used one and is well described in Sañudo (2009) [31]. The animals were restrained by the head following standard procedures. Measurements were taken by two trained students who participated in data collection on the farms. Measurements were taken directly from the animal with a measuring tape (Ovny, Inalmet, Bta. CO) graduated in centimeters. Body weight was measured with a hanging digital scale (IF1976, Bta. CO). One student took the measurement and the other recorded the value. Measurements were taken in duplicate. Age was obtained from the information available in each animal’s record. The measurements were taken between April and November 2025.

The linear body variables taken in the Santandereana Creole goat, including body weight (PC), were: body length (LCO), thoracic perimeter (PT), withers height (ALC), bicostal width (DB), dorso-sternal diameter (DD), thorax width (ANT), chest width (ANESP), height ground-sternum (ALES), cannon perimeter (PCA), cannon length (LCÑ), rump height (ALGR), rump width (AGR), rump length (LGR), height to tail base (ANC), height to hock (ACO), head width (ACA), head length (LCA), face width (AC), face length (LC), skull length (LCR), skull width (ACR), horn perimeter (right and left respectively, PCD, PCI), horn length (right and left respectively, LCD, LCI), ear length (right and left respectively, LOD, LOI), and ear width (right and left respectively, AOD, AOI). Paired measurements (those referred to horns and ears) were taken to assess asymmetries.

From these linear values, a set of 16 indexes (7 ethnological and 9 functional) was derived, as described in the literature [17,32,33,34] (Table 1).

### 2.4. Statistical Analysis

A Mann–Whitney test assessed right–left differences in horns (length and perimeter) and ears (length and width). A one-way linear PERMANOVA model, using Gower distances, allowed for testing differences between sexes. A principal component analysis (PCA) from a var-covar matrix was done to detect most discriminative traits. Finally, a one-way linear ANCOVA model, using loadings on PC1, was done to compare allometric tendencies between sexes.

The statistical analyses were performed using PAST v. 4.17c [35]. The confidence level was set at 95%.

## 3. Results

**Figure 2:**
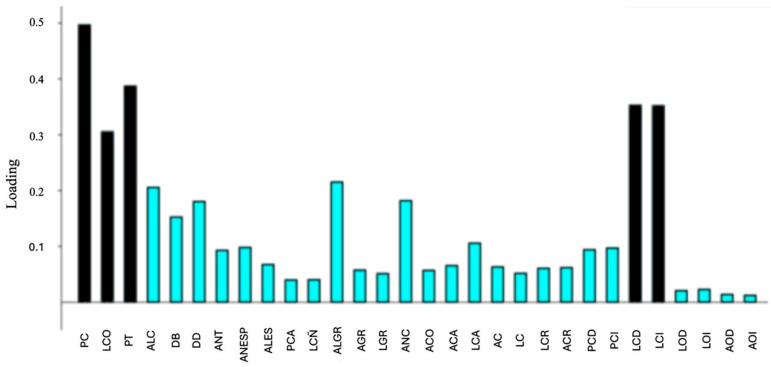
Loading on first principal components of PCA, which explained 67.69% of the total observed variance. Differences were observed in size, with most discriminative values being those referred to body (PC = body weight, kg; LCO = body length, cm; PT = thoracic perimeter, cm) and to horns (LCD = right horn length, cm; LCI = left horn length, cm). *y*-axis: loading values; *x*-axis: zoometric variables. See text for acronyms.

Table 2 reflects main descriptive statistics of the zoometric variables for each sex, and Table 3 the corresponding indexes. Most of the variables were not normally distributed. No right–left differences appeared in horns and ears. There were differences between sexes for lineal traits (*F* = 4.302; *p* = 0.0101) as well as for indexes (*F* = 2.284; *p* = 0.0364). First two principal components of PCA explained 75.6% of the total observed variance (PC1 + PC2 = 67.69% + 7.91%). Differences were observed in size, with most discriminative values being those referred to body (PC, LCO and PT) and to horns (LCD and LCI) (Figure 2). Indexes corroborated the bigger size among males, especially in relation to skull width, rump length, thorax depth and body weight.

**Figure 3:**
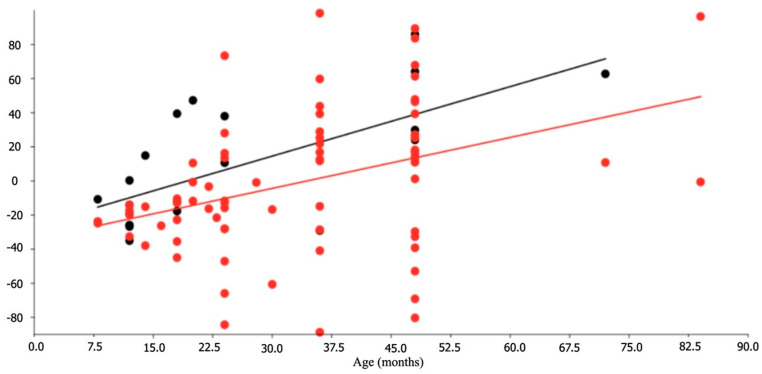
Allometric trends for males and females of Santandereana Creole goat breed (males: upper line, n = 23, 8–72 months of age; females n = 76, 8–84 months of age). Dependent variables (*Y*-axis) were individual loadings on principal component 1.

There were allometric differences between sexes (*F* = 4.317; *p* = 0.040), with the development of males being clearly faster (Figure 3). The following regression equations allowed for the prediction of animal growth during the studied period:

BW = 16.93 + (0.678 E) (males) (r^2^ = 0.573; *p* << 0.001)
BW = 22.48 + (0.234 E) (females) (r^2^ = 0.338; *p* << 0.001)

where

BW = Body weight, kg
E = Age of the animal, months

## 4. Discussion

### 4.1. Body Measurements and Sexual Differences

In this study, significant differences in growth between sexes were observed in body size, with the most discriminating values being in body weight, body length, and thoracic perimeter. A significant difference was also observed in horn length (right/left) (Table 2, Figure 2).

Males presented the highest values for all five of the traits. These size-related variables were also discriminatory in Sokoto goat populations in West Africa [11]. In the Motilona Creole goat of Colombia, body weight, thoracic perimeter, and body length were also discriminatory and biased toward males, although the difference in the latter was not significant [36]; similarly, no sexual differentiation was observed in body weight in goats from Costa Rica [37]. Horn length was a distinguishing trait in the comparison between two African goat breeds [11]; although the researchers did not account for sexual differentiation, the means were lower than those reported for the Santandereana goat. In Maasai goats, body length and thoracic perimeter were greater in males [38], while in Chilean goats, thoracic perimeter and body weight showed differences between sexes, with lower values for females [39], differing from the results for the Santandereana Creole goat.

In Nicaraguan goats, thoracic perimeter, length, and body weight showed lower values in males, without statistical difference, but with a higher level of variation [40]. These data contrast with those reported for the Santander Creole goat in the present study. A characterization study on Pastoreña Creole goats of Mexico showed a male-biased sexual difference for all the morphometric measurements analyzed [41]. Cuban Creole goats also show sexual differentiation in thoracic perimeter and body length, biased towards males [42].

Biometric studies in West African dwarf goats reported that live weight and body measurements showed sexual differentiation, with higher values in females [43], while in Nigeria, also in dwarf goats, sex did not show a significant influence on all the morphometric traits studied, although females were superior in the body variables analyzed [44,45]. In Creole goats from Puebla, Mexico, the females showed a larger thoracic perimeter and the males greater body length, without significant sexual differentiation [46], which differs from that reported for the Santandereana Creole goat.

In the characterization of the Pampeana goat, the difference between sexes was significant for body length; in the kids, it was greater in females, while in young and adult animals, the values were higher in males [47]. Another study conducted by the same author found sex differences in body length, weight, and thoracic perimeter, biased toward males and with a wide range, indicating greater selection in some farms or the presence of different regional ecotypes [48].

Allometric growth reflected differences between sexes, with males showing faster development. This result differs from that of the Chusca Lojana goat, where faster growth was observed in females until two years of age, after which they begin to give birth and growth slows due to lactation [1]. In the characterization of goats in Kenya, it was observed that thoracic perimeter increased with age [49], but with lower values than those recorded in the Santandereana Creole goat. Studies on Canary Island goats have confirmed that sex has a highly significant effect on weight and body measurements, always greater in males. The same study showed no sexual differentiation in thoracic perimeter during the first three months of life, indicating that chest growth is low during this period [50].

In the morphometric characterization of dwarf goats in Ghana, it was shown that age and agroecological zone significantly influence most body traits, including ear length [13]. The findings of sexual or non-sexual differentiation within the same breed imply that there may be inherent differences in body weight and biometric measurements, and it could be due to an adverse environment in terms of ambient temperature and poor nutrition in which the goats are raised [45].

A significant difference was also observed in the growth of horn length (right/left), biased towards males in both horns. Similar results have been observed in mountain goats (*Oreamnos americanus*), where males exhibit longer horns and faster growth during the first 18 months of life, showing sexual dimorphism in size [51]. Another study comparing mountain goats from two different areas showed that females exhibited greater growth, indicating that the environment has a distinct effect on horn growth in both sexes [52]. Horn growth is an indicator of habitat quality and is negatively affected by lactation. It is unclear whether there is an individual benefit to developing long horns [51], and it has been suggested that horn length may decrease with increasing population densities [53].

The presence of horns represents adaptive responses to different ecological niches and selective pressures, and is considered an indicator of genetic fitness and breed quality [54]. Horns have evolved as adaptations for defense, intraspecific competition, and hierarchies. Horn growth is influenced by environmental, genetic, and hormonal factors [55]. These characteristics reflect the importance of the Santandereana Creole goat, which grows and lives in an adverse environment, yet has gained economic and productive importance for producers in the region. Horns, as secondary sexual characteristics, are biologically important because they can positively affect male fitness through sexual selection. Therefore, further research should be conducted on horn growth in the Santanderean Creole goat.

Growth, sex determination and breeding are interconnected with genetic and hormonal factors directing sexual differentiation in the embryo. While chromosomes (XY or ZW) usually determine sex, hormones drive the development of male or female traits; different hormone levels during gestation and puberty contribute to sexual dimorphism [56]. Growth rates often differ between sexes, with males often being larger, as is the case in the Santandereana Creole goat. Differences may reflect sexual variations in the development of skeletal maturity. In small ruminants, the onset of puberty is determined by age, weight, photoperiod and breed [57]. There is scarce information on reproductive basic patterns in the Santandereana Creole goat and information from most published studies is valid for specific locations.

For the conservation of native genetic resources, morphological characterization of animals through the expression of their quantitative traits is necessary [34,58]. Morphometric characterization is an alternative that facilitates the conservation of the diversity of native or creole animals and helps to establish breed inventories and monitor endangered species [59]. It has been reported that body morphometric traits are heritable and can be used for breeding programs [60]; therefore, body measurements could be applied to defined selection patterns, considering the zootechnical orientation [61]. Morphometric measurements have been shown to be useful for differentiating the size and shape of animals [62] and could be considered an indicator for describing a breed [34].

### 4.2. Body Indexes and Sexual Differences

The body indexes obtained in the present study confirmed sexual differentiation in growth, showing the most discriminating values related to skull width (cephalic index), rump length (pelvic index, longitudinal pelvic index), chest depth (relative thoracic depth index), and body weight (compactness index). Females showed higher values in the longitudinal pelvic index and relative thoracic depth, and males in the pelvic, cephalic, and compactness indexes. Sexual differentiation for the same indexes was also reported for goats in Chile, with values biased towards females in the pelvic index and longitudinal pelvic indices [39]. In general, the values reported in Chilean goats show different variation compared to those reported for the Santandereana Creole goat.

Pelvic index (rump width/rump length × 100). This index expresses the structure of the rump, whether it is square, short, or long, and is related to reproductive aptitude. The higher the value, the wider the pelvis is than long, or vice versa, or square. The rump can be classified as concavelinear (>100) or convexilinear (<100) [32,34]. The index value for the Santandereana Creole goat (93.9) shows a predominance of width over length, which indicates an ease of giving birth, a characteristic relevant in creole animals. This index value was similar to that recorded in goats from Nicaragua [40], and lower than that reported in goats from Chile, where, moreover, the rate increased with age in females and decreased in males [39]. The Santandereana Creole goat presented a pelvic index higher than that reported for the Pampeana goat [47]. The Santandereana Creole goat tends to have a square rump with a convex line.

Longitudinal pelvic index (rump length/height at the withers × 100). Also called longitudinal ilio-ischiatic index, it is used to estimate the livestock aptitude of a breed. An index that does not exceed 37 is an appropriate indicator in a meat-producing animal [34], which indicates that the Santander goat is an animal with a predisposition for meat production. The same trend was observed in the Pampeana goats [47], and goats from Chile [39], México [61], and Nicaragua [40].

Relative depth of thoracic index (dorsal-sternal diameter/height at the withers × 100). Its value indicates the depth or height of the chest in relation to the height at the withers, showing animals with good or low productive capacity. A value greater than 50 is indicative of animals with a tendency toward the meat-type phenotype [34], and in a certain way indicates the length of the limbs or the distance between the chest and the ground [40]. Animals with higher values (>50) have kinetic capabilities suitable for extensive grazing in hot climates, as the body stays further away from the solar radiation emitted by the ground [63]. According to the value of this index, the Santandereana Creole goat is oriented towards meat production. Lower indexes were recorded in Pampeana goats [47], Chile [39] and Nicaragua [40]. According to the index description, the Santanderean Creole goat is a dolichomorphic animal.

Compactness index (body weight/height at the withers × 100). This index is also referred to as “relative weight.” The index shows whether an animal is heavy or compact or slightly compact, and values greater than 100 are a reference for animals with meat aptitude [33,34]. The Santandereana Creole goat showed an index with values below 100, indicating that they are not compact; however, they tend to be meat producers, and the males register a higher relative weight than the females.

Cranial index (skull width/skull length × 100). It is considered an ethnological index, and a value above 80 reveals that an animal has skulls that are wider than long [64]. Although the variables skull width and length did not show discriminating power between the sexes, it did appear in the cranial index, which demonstrates the importance for ethnological diagnosis [19], or in our case, for sexual differentiation. Furthermore, the shape of the head is not influenced by environmental factors or management, so the cranial index is of interest for racial–ethnological diagnosis [18,65]. According to the index reported in this study for the Santandereana Creole goat, the males (CI = 200.3) have wider and longer skulls than the females (CI = 184.6), so they are considered brachycranial animals.

We recommend continuing to take body measurements and body weight of the goats until they reach adult weight, to complete the growth curves and to associate them with productive traits and the measurements obtained at an early age, which would allow for more accurate estimates of the animals at different ages. This allows for earlier breeding decisions and increases the annual rate of genetic improvement. Prediction regression equations of animal growth during the studied period for males and females are finally provided.

## 5. Conclusions

In the study of the Santandereana Creole goat, body weight, body length, thoracic perimeter, and horn length reflected sexual dimorphism of size. The indexes confirm that the Santandereana Creole goat is an animal with a tendency for meat production, with larger males, a square rump with a convex line, dolichomorphics, and brachycephalic and brachycranial features due to the shape of the head. Detected differences may reflect variations in the development of skeletal maturity. These parameters can be considered of interest for preserving the breed and establishing genetic improvement programs and adaptation to arid regions.

## Figures and Tables

**Table 1:** Ethnological and functional indexes evaluated in the Santandereana Creole goat of Colombia.

Indexes	Assessment
Corporal index (CI): body length/thoracic perimeter × 100	ethnological
Thoracic index (TI): bicostal width/dorso-sternal diameter × 100	ethnological
Cephalic index CI): head width/head length × 100	ethnological
Pelvic index (PI): rump width/rump length × 100	ethnological
Proportionality index (PI): withers height/body length × 100	functional
Metacarpo-thoracic index (dactilo-thoracic) (MCI): cannon perimeter/thoracic perimeter × 100	functional
Relative depth of thoracic index (RDI): dorso-sternal diameter/withers height × 100	functional
Dactilo-costal index (DCI): cannon perimeter/thorax width × 100	functional
Transversal pelvic index (TPI): rump width/withers height × 100	functional
Longitudinal pelvic index (LPI): rump length/withers height × 100	functional
Relative thickness of cannon index (RTI): cannon perimeter/withers height × 100	functional
Cranial index CRI): skull width/skull length × 100	ethnological
Facial index (FI): face width/face length × 100	ethnological
Compactness index (COI): body weight/withers height × 100	functional
Cannon load index (CLI): cannon perimeter/body weight × 100	functional
Anamorphosis index (AI): (thoracic perimeter)^2^/withers height	ethnological

**Table 2:** Descriptive statistics of the zoometric variables for males (n = 23; 8–72 months of age) and females (n = 76; 8–84 months of age) of Santandereana Creole goat breed. See text for acronyms. Lineal measurements expressed in cm, except for body weight (PC) in kg.

Body Variable	Sex	Min	Max	Mean ± SD	Median	CV (%)
Body weight (PC)	M	18.0	72.0	34.2 ± 16.01 a	29.0	46.9
	F	17.0	45.0	30.5 ± 6.44 b	31.0	21.1
Body length (LCO)	M	53.0	91.0	66.9 ± 10.42 a	65.0	15.6
	F	51.0	77.0	64.6 ± 5.24 b	65.0	8.1
Thoracic perimeter (PT)	M	60.5	97.0	73.4 ± 11.96 a	70.0	16.3
	F	58.0	88.0	73.8 ± 6.72 b	75.0	9.1
Withers height (ALC)	M	52.0	77.1	62.8 ± 6.54	62.5	10.4
	F	52.5	75.4	62.1 ± 4.00	62.2	6.4
Bicostal width (DB)	M	23.3	40.0	30.1± 4.68	29.1	15.6
	F	25.0	39.0	30.8 ± 3.20	30.2	10.4
Dorso-sternal diameter (DD)	M	26.0	50.0	34.9 ± 6.25	34.5	17.9
	F	27.0	42.5	34.9 ± 3.04	35.0	8.7
Thorax width (ANT)	M	13.0	24.0	16.8 ± 3.37	16.0	20.1
	F	10.0	25.0	15.8 ± 2.96	15.2	18.7
Chest width (ANESP)	M	12.0	30.0	17.2 ± 4.48	17.0	26.0
	F	10.2	23.0	15.5 ± 3.27	15.5	21.0
Height ground-sternum (ALES)	M	30.0	45.0	34.8 ± 3.74	34.0	10.7
	F	26.0	41.8	34.2 ± 2.70	34.0	7.9
Cannon perimeter (PCA)	M	6.5	12.0	8.8 ± 1.39	8.5	15.7
	F	7.0	12.0	8.2 ± 0.83	8.0	10.1
Cannon length (LCÑ)	M	15.0	22.5	18.1 ± 1.97	18.0	10.9
	F	14.0	21.5	17.8 ± 1.51	18.0	8.5
Rump height (ALGR)	M	52.0	84.0	64.8 ± 7.49	65.3	11.6
	F	55.5	75.0	63.8 ± 3.93	63.5	6.2
Rump width (AGR)	M	9.2	19.0	14.0 ± 2.60	14.0	18.6
	F	9.0	20.0	13.9 ± 2.51	14.0	18.0
Rump length (LGR)	M	11.9	18	14.9 ± 1.67	14.5	11.2
	F	12.0	17.5	14.9 ± 1.15	15.0	7.7
Height to tail base (ANC)	M	43.0	70.0	55.8 ± 7.20	56.3	12.9
	F	44.0	73.0	54.4 ± 4.00	54.5	7.4
Height to hock (ACO)	M	22.0	30.0	25.6 ± 2.23	25.0	8.7
	F	20.0	29.0	25.0 ± 1.81	25.0	7.3
Head width (ACA)	M	10.0	18.0	12.9 ± 2.08	12.5	16.1
	F	9.2	18.8	12.0 ± 1.58	12.0	13.1
Head length (LCA)	M	21.0	39.5	27.6 ± 4.57	26.5	16.6
	F	20.0	31.0	26.3 ± 2.72	26.5	10.4
Face width (AC)	M	10.0	21.0	13.7 ± 2.57	13.2	18.8
	F	11.0	16.0	13.3 ± 1.15	13.0	8.6
Face length (LC)	M	12.0	21.1	15.8 ± 2.19	16.0	13.9
	F	11.5	19.0	15.4 ± 1.86	15.1	12.0
Skull length (LCR)	M	8.0	19.0	11.9 ± 3.12	12.0	26.1
	F	7.5	16.0	11.5 ± 2.37	11.1	20.7
Skull width (ACR)	M	15.0	32.7	22.7 ± 4.36	22.0	19.2
	F	14.0	25.0	20.2 ± 1.70	20.2	8.4
Right horn perimeter (PCD)	M	7.0	21.0	12.3 ± 3.67	12.3	29.9
	F	5.8	12.0	9.2 ± 1.21	9.5	13.1
Right horn length (LCD)	M	6.5	58.5	21.9 ± 12.01 a	20.0	54.7
	F	6.0	28.5	15.9 ± 4.05 b	16.0	25.5
Left horn perimeter (PCI)	M	7.0	21.5	12.2 ± 3.67	12.2	30.0
	F	7.0	13.5	9.3 ± 1.18	9.2	12.7
Left horn length (LCI)	M	7.5	56.3	21.9 ± 11.85 a	20.0	54.2
	F	5.5	29.4	16.1 ± 4.16 b	16.1	25.9
Right ear length (LOD)	M	13.0	20.0	15.8 ± 1.69	16.0	10.7
	F	12.4	18.0	15.6 ± 1.30	16.0	8.4
Right ear width (AOD)	M	6.0	8.3	7.0 ± 0.73	7.0	10.5
	F	5.5	8.0	6.9 ± 0.51	7.0	7.3
Left ear length (LOI)	M	13.0	20.0	15.7 ± 1.44	16.0	9.2
	F	12.2	18.0	15.4 ± 1.38	15.9	8.9
Left ear width (AOI)	M	6.0	8.0	7.1 ± 0.65	7.0	9.1
	F	5.5	8.0	6.9 ± 0.52	7.0	7.6

M: Males; F: Females; Min: Minimum; Max: Maximum; SD: Standard Deviation; CV: Coefficient of Variation. Means with different letters between sexes differ statistically (*p* < 0.05).

**Table 3:** Body indexes for males (n = 23; 8–72 months of age) and females (n = 76; 8–84 months of age) of Santandereana Creole goat breed.

Body Indexes (%)	Sex	Min	Max	Mean ± SD	Median	CV (%)
Corporal index (CI)	M	77.1	103.2	91.4 ± 5.9	93.2	6.5
	F	71.3	112.1	88.0 ± 8.4	88.8	9.5
Thoracic index (TI)	M	76.1	100.0	86.7 ± 5.6	86.6	6.5
	F	76.3	110.3	88.2 ± 6.2	87.0	7.0
Cephalic index CI)	M	37.5	66.7	50.2 ± 7.7	50.0	15.4
	F	35.5	63.8	50.9 ± 5.6	51.7	11.1
Pelvic index (PI)	M	70.6	115.2	93.9 ± 13.4 a	93.8	14.3
	F	66.7	120.9	92.9 ± 13.4 b	93.8	14.4
Proportionality index (PI)	M	78.0	115.7	94.9 ± 8.7	94.2	9.1
	F	82.4	114.5	96.6 ± 7.9	97.2	8.1
Metacarpo-thoracic index (dactilo-thoracic) (MCI)	M	10.4	14.0	12.1 ± 0.9	12.2	7.6
	F	9.3	15.0	11.1 ± 1.0	10.9	8.7
Relative depth of thoracic index (RDI)	M	43.3	69.0	55.3 ± 6.0 a	56.1	10.9
	F	46.6	66.2	56.2 ± 4.1 b	56.1	7.4
Dactilo-costal index (DCI)	M	44.8	63.9	53.4 ± 6.0	53.3	11.3
	F	36.0	71.4	52.8 ± 7.3	53.1	13.8
Transversal pelvic index (TPI)	M	15.7	27.6	22.3 ± 3.8	23.1	16.9
	F	15.2	30.0	22.4 ± 3.6	22.4	16.1
Longitudinal pelvic index (LPI)	M	21.3	26.2	23.7 ± 1.2 a	23.7	5.3
	F	20.7	29.2	24.1 ± 1.8 b	23.9	7.4
Relative thickness of cannon index (RTI)	M	11.5	16.9	14.1 ± 1.4	13.8	10.0
	F	10.5	19.3	13.2 ± 1.2	13.1	9.4
Cranial index CRI)	M	97.2	363.3	200.3 ± 55.5 a	186.7	27.7
	F	118.8	285.0	184.6 ± 41.7 b	186.1	22.6
Facial index (FI)	M	62.5	116.7	87.3 ± 13.5	87.5	15.5
	F	63.2	112.9	86.9 ± 10.6	87.3	12.1
Compactness index (COI)	M	32.7	101.4	52.9 ± 19.3 a	44.8	36.5
	F	31.3	67.2	48.9 ± 9.0 b	48.6	18.4
Cannon load index (CLI)	M	16.5	44.4	29.0 ± 7.9	29.6	27.3
	F	20.0	41.2	27.8 ± 5.4	26.6	19.4
Anamorphosis index (AI)	M	60.3	132.5	86.4 ± 21.2	79.7	24.5
	F	54.3	118.2	88.1 ± 13.3	89.6	15.1

M: Males; F: Females; Min: Minimum; Max: Maximum; SD: Standard Deviation; CV: Coefficient of Variation. Means with different letters between sexes differ statistically (*p* < 0.05).

## Data Availability

The data presented in this study are available on request from the the second author.the data are not publicly available due to we are going to carry out further analysis.
